# *Prognathodesgeminus*, a new species of butterflyfish (Teleostei, Chaetodontidae) from Palau

**DOI:** 10.3897/zookeys.835.32562

**Published:** 2019-04-04

**Authors:** Joshua M. Copus, Richard L. Pyle, Brian D. Greene, John E. Randall

**Affiliations:** 1 Hawai‘i Institute of Marine Biology, 46-007 Lilipuna Road, Kāne‘ohe, Hawai‘i 96744, USA Hawai‘i Institute of Marine Biology Kaneohe United States of America; 2 Bernice Pauahi Bishop Museum, 1525 Bernice Street, Honolulu, Hawai‘i 96817, USA Bernice Pauahi Bishop Museum Honolulu United States of America; 3 Association for Marine Exploration, 47-224 Kamehameha Highway, Kāne‘ohe, Hawai‘i 96744, USA Association for Marine Exploration Kāne‘ohe United States of America

**Keywords:** Closed-circuit rebreather, Mesophotic Coral Ecosystem, Micronesia, systematic ichthyology

## Abstract

A new species of the butterflyfish genus *Prognathodes* (Chaetodontidae) is described from two specimens collected at a depth of 116 m off Ngemelis Island, Palau. *Prognathodesgeminus***sp. n.** is similar to *P.basabei* Pyle & Kosaki, 2016 from the Hawaiian archipelago, and *P.guezei* (Maugé & Bauchot, 1976) from the western Indian Ocean, but differs from these species in the number of soft dorsal-fin rays, size of head, body width, and body depth. There are also subtle differences in life color, and substantial differences in the mtDNA cytochrome oxidase I sequence (d ≈ 0.08). Although genetic comparisons with *P.guezei* are unavailable, it is expected that the genetic divergence between *P.guezei* and *P.geminus* will be even greater than that between *P.geminus* and *P.basabei*. It is named for the strikingly similar color pattern it shares with *P.basabei*.

## Introduction

The butterflyfish genus *Prognathodes* Gill, 1862 (type species *Chelmopelta* Günther, 1860 = *Chaetodonaculeatus* Poey, 1860) currently includes twelve valid species: seven from the Atlantic [*P.aculeatus* (Poey, 1860), *P.aya* (Jordan, 1886), *P.brasiliensis* Burgess, 2001, *P.dichrous* (Günther, 1869), *P.guyanensis* (Durand, 1960), *P.marcellae* (Poll, 1950), and *P.obliquus* (Lubbock & Edwards, 1980)], two from the tropical eastern Pacific [*P.falcifer* (Hubbs & Rechnitzer, 1958), *P.carlhubbsi* Nalbant, 1995], one from the Hawaiian Islands [*P.basabei* Pyle & Kosaki, 2016], one from the central Indian Ocean and western Pacific [*P.guyotensis* (Yamamoto & Tameka, 1982)], and one from the western Indian Ocean [*P.guezei* (Maugé & Bauchot, 1976)], most of which are associated with deep coral-reef environments known as Mesophotic Coral Reef ecosystems (MCEs; 30–150 m; Hinderstein et al. 2010).

Several individuals of a butterflyfish with dark bars resembling both *P.guezei* from the Western Indian Ocean and a species from the Hawaiian Islands that was later described as *P.basabei* were recorded on video at depths in excess of 110 m during a series of submersible dives in Palau conducted by Patrick L Colin and Lori Bell Colin in 2001. In April 2007, while conducting a series of deep exploratory dives off Ngemelis Island, Palau (Republic of Belau) in the Caroline Islands, authors Pyle and Greene collected two specimens of this unidentified *Prognathodes* at a depth of 116 m. The fish were encountered among a patch of limestone rubble at the base of a prominent limestone outcropping.

Based on an examination of a combination of morphological and genetic characters of the Palauan specimens, in comparison to six specimens of *P.basabei* and two specimens of *P.guezei* (the two species that most closely resemble the Palauan specimens), we herein describe the new species as *Prognathodesgeminus*.

## Materials and methods

Two unknown fish of the genus *Prognathodes* were collected with hand nets during deep dives using mixed-gas, closed-circuit rebreathers off of Ngemelis Island (7.13791N, 134.22181E), at a depth of 116 m. In order to capture the live coloration, the specimens were brought to the surface alive, euthanized, and immediately photographed. A tissue sample was taken from each specimen prior to being placed in formalin. Methods of counts and measurements follow Pyle and Kosaki (2016).

Head length, depth of body, width of body, snout length, predorsal length, preanal length, length of dorsal-fin and anal-fin bases, orbit diameter, interorbital width, caudal peduncle depth, and lengths of fin spines and rays are expressed as percent of standard length (as SL). Counts and measurements for the paratype, if different from the holotype, are presented in parentheses after the value for the holotype.

The holotype has been deposited in the fish collection at the Bernice Pauahi Bishop Museum, Honolulu (**BPBM**), and the paratype has been deposited at the US National Museum of Natural History, Washington, DC (**USNM**).

Total genomic DNA was extracted using the ‘HotSHOT’ protocol (Meeker et al. 2007). A 533-base pair fragment of the mtDNA cytochrome c oxidase 1 (CO1) region was amplified using primers from Baldwin et al. (2009). Polymerase chain reaction (PCR) was performed in a 15 μl reaction containing 7.5 μl BioMix Red (Biolone Inc, Springfield, NJ, USA), 0.2 μM of each primer, 5–50 ng template DNA, and nanopure water (Thermo Scientific Barnstead, Dubuque, IA, USA) to volume. PCR cycling parameters were as follows: initial 95 °C denaturation for 10 min followed by 35 cycles of 94 °C for 30 sec, 55 °C for 30 sec, and 72 °C for 30 sec, followed by a final extension of 72 °C for 10 min. PCR products were visualized using a 1.5% agarose gel with GelStar (Cambrex Bio Science Rockland, Inc., Rockland, MA, USA) and then cleaned by incubating with 0.75 units of Exonuclease and 0.5 units of Shrimp Akaline Phosphate (ExoSAP; USB, Cleveland, OH, USA) per 7.5 μl of PCR product for 30 min. at 37 °C followed by 85 °C for 15 min. Sequencing was conducted in the forward and reverse direction using a genetic analyzer (ABI 3730XL, Applied Biosystems, Foster City, California) at the ASGPB Genomics Sequencing Facility at the University of Hawai‘i at Mānoa. The sequences were aligned, edited and trimmed to a common length using Geneious Pro v.6.1.6 DNA analysis software (Biomatters. http://www.geneious.com/). CO1 haplotypes were deposited in GenBank and the Barcode of Life Database (BOLD).

## Taxonomy

### Prognathodes geminus sp. n.

Taxon classificationAnimaliaPerciformesChaetodontidae

http://zoobank.org/BEAB3B7F-B4B1-44F7-8181-2FF950CA466A

Figures 1 , 2 , 3

#### Type Locality.

Caroline Islands; Palau; Ngemelis Island, northwest side, “Blue Holes” (7.13791°N, 134.22181E), 116 m.

#### Holotype.

BPBM 40857, GenBank MG895424, Barcode of Life PROGE001-17, 73.1 mm SL, Caroline Islands; Palau; Ngemelis Island, northwest side, “Blue Holes”, 7.13791N, 134.22181E; 27 April 2007; around limestone rock outcrop with limestone rubble; 116 m; hand nets; RL Pyle.

#### Paratype.

USNM 440390, GenBank MG895423, Barcode of Life PROGE002-17, 1:70.0 mm SL, same location, habitat, collecting method, and date as holotype, BD Greene.

#### Diagnosis.

A species of *Prognathodes* (sensu Smith et al. 2003) distinguished by the following combination of characters: dorsal-fin soft rays 17–19; anal-fin soft rays 15; head 2.48–2.49 in SL; body depth 1.71–1.76 in SL; pelvic-fin spine length 4.18–4.46 in SL; body color white with three broad dark brown bands, the first intersecting the eye.

#### Description.

Dorsal fin XIII,17 (19), last soft ray branched to base; anal fin III,15, last soft ray branched to base; pectoral-fin rays 15; pelvic-fin rays I,5; principal branched caudal rays 15; pored lateral-line scales 26 (27); scale rows above lateral line to origin of dorsal fin 11 (10); scale rows below lateral line to origin of anal fin 21 (20); gill rakers on upper limb 5 (6), on lower limb 10; vertebrae 24.

Body deep, depth 1.71 (1.76) in SL, and compressed, width 4.36 (3.2) in depth; head length 2.49 (2.48) in SL; snout produced, its length 2.37 (2.52) in head; orbit diameter 3.30 (3.53) in head; interorbital slightly convex, least bony width 4.59 (4.78) in head; least depth of caudal peduncle 4.59 (4.78) in head.

Mouth small, upper jaw 2.37 (2.52) in head, slightly diagonal, the gape forming an angle of about 20° to the horizontal, upper jaw slightly protruding; teeth in jaws densely setiform, longest 7.8 in orbit diameter; nostrils anterior to eye horizontally in line with center of iris, the anterior in a short membranous tube with a well-developed posterior flap, the posterior slightly larger, ovate, with a low fleshy rim. Lower edge of lacrimal smooth; margin of preopercle finely serrate; margins of other opercular bones smooth.

Lateral line forming a broad arc, ending below the base of third to fifth soft dorsal rays and within the second black band of the body. Scales ctenoid, moderately large on body except for chest and near origins of dorsal and anal fins, where small; head fully scaled except anterior portions of both jaws and around nostrils, scales on the head small; scales on fleshy sheath surrounding base of dorsal and anal fins moderately large anteriorly and proximally, reducing in size posteriorly and distally; scales on caudal peduncle and covering base of caudal fin small.

Origin of dorsal fin slightly anterior to upper end of gill opening, its base 1.5 (1.51) in SL; first dorsal-fin spine shortest, its length 4.03 in head; second dorsal-fin spine 1.85 (1.88),in head; fourth dorsal-fin spine longest, its length 1.18 (1.26) in head; third dorsal-fin spine nearly as long as fourth, its length 1.23(1.28) in head; fifth dorsal-fin spine shorter, its length 1.28 (1.29) in head; dorsal-fin spines progressively shorter posteriorly, the last 1.96 (1.89) in head; membranes between anterior dorsal-fin spines deeply incised, progressively less so posteriorly; first dorsal-fin soft ray the longest, approximately same length as last dorsal-fin spine, 1.87 (broken in paratype) in head, dorsal-fin soft rays progressively shorter posteriorly; first anal-fin spine shortest, its length 2.67 (2.56) in head; second anal-fin spine longest, its length 1.33 (1.38) in head; third anal-fin spine 1.44 (1.53) in head; first anal-fin soft ray longest, its length 1.40 (damaged in paratype) in head, anal-fin soft rays progressively shorter posteriorly; caudal fin damaged; pectoral fins damaged; pelvic spine 1.68 (1.8) in head; first soft ray of pelvic fin broken in both specimens.

**Figure 1.**
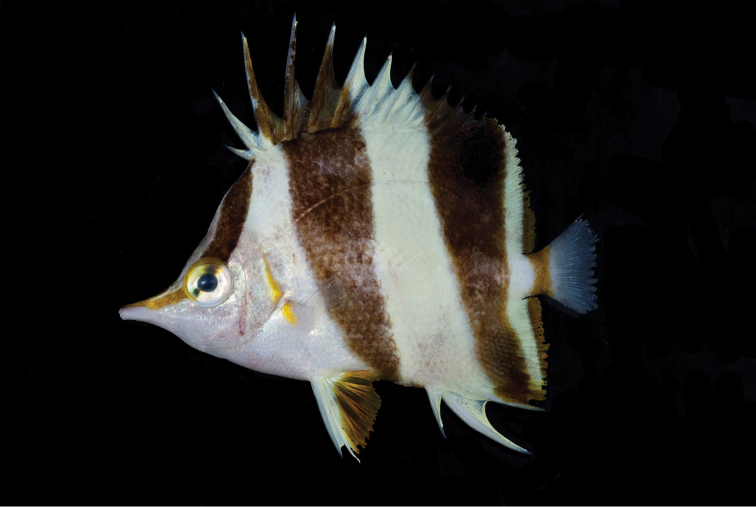
Holotype of *Prognathodesgeminus* (BPBM 40857), collected at a depth of 116 m at Palau. Photograph by RL Pyle.

**Figure 2.**
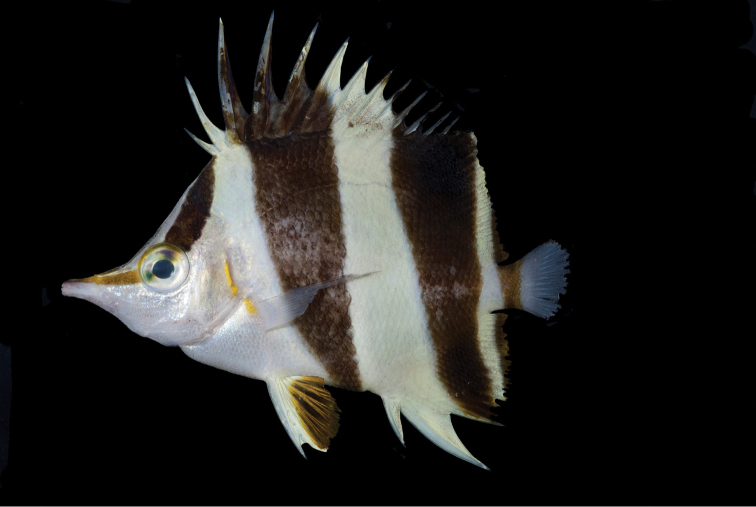
Paratype of *Prognathodesgeminus* (USNM 440390), collected at a depth of 116 m at Palau. Photograph by RL Pyle.

Color in life as in Figures 1 and 2. Body white with three dark brown bands, the first beginning narrowly at origin of dorsal fin, curving slightly as it passes, mostly one-half eye diameter in width, to eye; second band originating at tips of third to fifth dorsal spines and basal half of fifth spine, crossing body ventrally, progressively narrower below lateral line to end on mid-abdomen one-half eye diameter in width; third dark brown band originating from outer third of eighth dorsal spine and tip of first two soft rays of fin, passing obliquely, equal in width, to lateral line, then narrowing to half that width at mid-base of soft portion of anal fin, and crossing fin to end on outer half of third anal spine; a yellowish brown bar, one-half eye diameter in average width (narrowing gradually to mid-peduncle depth), across caudal peduncle and base of caudal fin; remainder of caudal fin whitish; a yellowish brown band of pupil width passing obliquely from eye, narrowing on its anterior half, to end at tip of snout; eye silvery white with an oblique dark brown band in alignment with nape band across eye, the dorsal part half-pupil width, and the ventral part one-half that; opercular membrane bright yellow; margin of soft portion of anal fin dark yellowish brown, about pupil width on last rays and membranes, progressively narrower to first rays; a similar terminal margin on dorsal fin; pectoral fins translucent with a semicircular light gray bar at base, preceded on upper half by a bright yellow bar. about half-pupil diameter in width, in alignment with yellow margin of operculum; spine and first ray of pelvic fins white, the white continuing progressively shorter on remaining rays that are dark orange-yellow, the membranes translucent dark yellowish.

Color in alcohol similar to life color, except body a uniform dull yellow, bands dark brown, and orange areas pale brown.

Morphometric data for selected characters of type specimens are provided in Table 1.

**Table 1.** Morphometric and meristic data for selected characters of type specimens of *Prognathodesgeminus*. Values of morphometric data (other than SL) are represented as % of SL.

Morphometrics	Holotype	Paratype
	BPBM 40857	USNM 440390
Standard length (SL) in mm	73.1	70.1
Body depth	58	57
Body width	13	13
Head length	40	40
Snout length	16	17
Orbit diameter	12.2	11.4
Interorbital Width	9.0	8.0
Predorsal length	46	42
Preanal length	72	71
Base of dorsal fin	67	66
Base of anal fin	30	30
Caudal Peduncle Depth	8.8	8.4
Pelvic Spine	23.9	22.4
Pelvic Fin	–	–
First Dorsal Spine length	10	10
Second Dorsal Spine length	22	21
Third Dorsal Spine length	33	31
Fourth Dorsal Spine length	34	32
Fifth Dorsal Spine length	31	31
Last Dorsal Spine length	21	21
Longest Dorsal Ray length	21	–
First Anal Spine length	15	16
Second Anal Spine length	30	29
Third Anal Spine length	28	26
Longest anal ray length	29	–
Caudal fin length	–	–
Pectoral fin length	–	–
**Meristics**		
Dorsal Spines	XIII	XIII
Dorsal rays	17	19
Anal Spines	3	3
Anal Rays	15	15
Pectoral Rays	15	15
Caudal Rays	22	22
Pored lateral line scales	26	27
Dorsal scale rows	11	10
Ventral scale rows	21	20
Gill rakers	5+10	6+10

#### Distribution.

*Prognathodesgeminus* is positively known only from Palau. However, individuals of what appear to be this species were collected by aquarium-fish collector Tim Bennett in the Coral Sea at a depth of 140 m (Fenton Walsh, pers. comm.), and video taken from a depth of about 120 m in New Caledonia (and reviewed by co-author Pyle) show what appears to be a similar fish. A similar species was recently described from the Hawaiian Islands (*P.basabei*), but numerous deep dives by the authors and others in regions between Palau and the Hawaiian Islands have not yielded any observations of this species, nor any other members of the genus *Prognathodes*.

#### Habitat.

**Figure 3.**
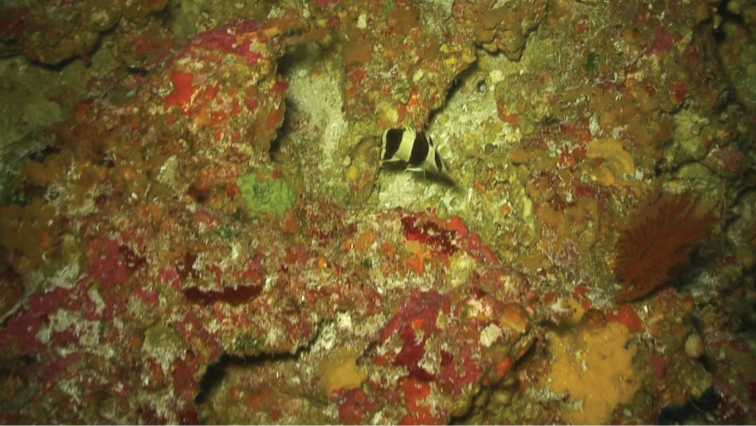
Holotype of *Prognathodesgeminus* in its natural habitat at a depth of 116 m off Ngemelis, Palau. Photograph by JL Earle.

Type specimens and other individuals observed from submersible by Patrick L Colin (pers. comm.) in Palau were seen in association with limestone outcroppings on steep slopes at depths of 110–150 m. The type specimens were collected in an area with broken limestone rubble (Figure 3).

#### Etymology.

We name this species *geminus*, Latin adjective for “twin”, in reference to its similarity in color to *P.basabei* from the Hawaiian Islands.

##### Morphological comparisons

**Figure 4.**
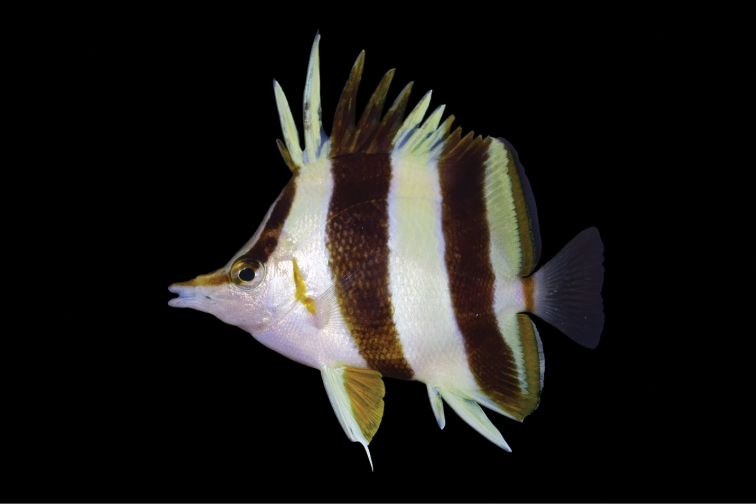
Holotype of *Prognathodesbasabei* (BPBM 41290), collected at a depth of 61 m off Pearl and Hermes Atoll, northwestern Hawaiian islands. Photograph by RL Pyle.

*Prognathodesgeminus* appears to be most similar in color and morphology to *P.basabei* collected at similar depths in the Hawaiian Archipelago. These two species differ from each other in number of dorsal-fin soft rays (17–19 for *geminus*, compared to 21–22 for *basabei*) and anal-fin soft rays (15 compared to 16–17). *P.geminus* has a larger head (2.48–2.49 in SL, compared to 2.63–2.80 in SL), its body more elongate, the depth 1.71–1.76 in SL, compared to 1.58–1.69 for *P.basabei*, more slender, the width 7.46–7.95 in SL compared to 6.40–7.04, and a shorter pelvic-fin spine (4.18–4.46 in SL, compared to 3.63–4.07 in SL)^1^ than the Palau species. The two species also differ in certain aspects of life color. The posterior edge of the first black band of *P.geminus* (Figures 1–3) ends at the origin of the first dorsal spine while it includes the first dorsal spine in *P.basabei* (Figure 4). The anterior edge of the second black band of *P.geminus* originates at the third dorsal-fin spine, whereas this band originates on the fourth dorsal-fin spine in *P.basabei*. Moreover, both of the dark bands on *P.geminus* are proportionally broader dorsally, tapering more substantially ventrally than in *P.basabei*. Also, the pale areas between dark bands are white in *P.geminus*, but pale yellow in *P.basabei*, and the orangish coloration on the pelvic fins and posterior margin of the soft dorsal and anal fins of *P.geminus* are much darker (brownish) than in *P.basabei*.

**Figure 5.**
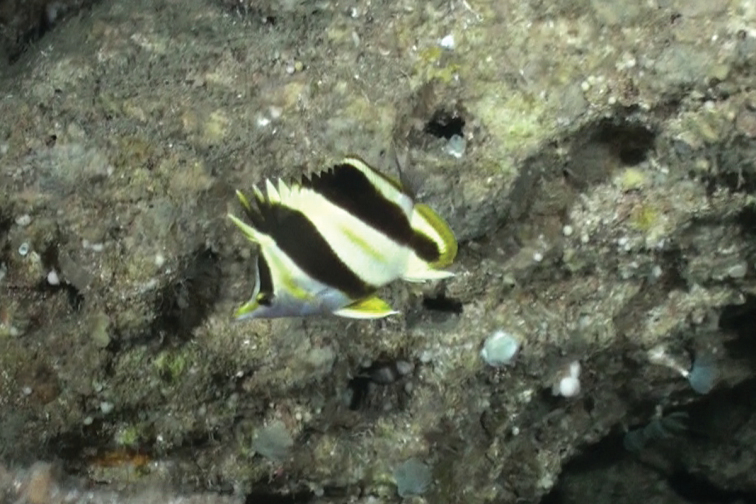
*Prognathodesguezei* at a depth of 117 m off Sodwana Bay, South Africa. Photograph by RL Pyle.

*Prognathodesgeminus* is also similar in color and morphology to *P.guezei* (Figure 5) from the western Indian Ocean. It differs from that species morphologically in number of dorsal-fin soft rays (17–19 for *geminus*, compared to 20 for *guezei*), body depth (1.71–1.76 in SL, compared to 1.87–1.95 in SL), and body width (7.46–7.95 in SL, compared to 7.16–7.27). There are also several differences in life color between the two species. In particular, *P.guezei* has more pronounced and discrete yellow bars on the body between the black bands, compared with more white in *P.geminus*. The two black bands on the body of *P.guezei* taper even more substantially than they do in the *P.geminus*. Also, the orangish coloration on the pelvic fins and posterior margin of the soft dorsal and anal fins of *P.guezei* are much paler and more yellowish than in *P.geminus*.

##### Genetic comparisons

A comparison of mtDNA COI sequences obtained from the holotype and paratype of *P.geminus* and from the holotype and two paratypes of *P.basabei* reveal 8% uncorrected sequence divergence, consistent with species-level divergences in other fish taxa (Johns and Avise 1998, Bowen et al. 2001, Bellwood et al. 2004, Fessler and Westneat 2007, Randall and Rocha 2009, Reece et al. 2010, Rocha 2004, Rocha et al. 2008). By contrast, the two specimens of *P.geminus* did not differ from each other genetically at COI, and the tree specimens of *P.basabei* differ only by 0.02%. While no tissue samples or DNA sequences have been reported for *P.guezei*, we expect that given the geographic distributions of *P.guezei* in the western Indian Ocean, and *P.geminus* and *P.basabei* in the Pacific Ocean, a much greater genetic divergence between *P.guezei* and the other two species likely exists.

## Discussion

Mixed-gas closed-circuit rebreather diving technology has revolutionized the exploration of the deeper depths of coral reef environments across the globe (Pyle 1996, 2000). *Prognathodesgeminus* is just one of many new fish species being discovered on MCEs and many more species are yet to be discovered and described. One of the more interesting aspects of this new species is the contrast between the strikingly similar color pattern it shares with *P.basabei*, against the substantial genetic differences. While genetic comparisons with *P.guezei* are not possible without appropriate tissue samples from that species, we expect that the genetic divergence between that species and *P.geminus* to be even greater than that between *P.geminus* and *P.basabei*. The disparity between the degree of difference in color relative to the genetic difference is striking in comparison to many species of butterflyfishes, which tend to have more substantial color differences relative to genetic differences (Hemingson et al. 2018).

*Prognathodesgeminus* is the thirteenth member of the genus. Given the proclivity for species of this genus to inhabit relatively poorly explored MCEs, as well as the stark genetic differences between *P.basabei* and its closest known relative, it would not be unusual for other species of this genus to be discovered elsewhere throughout the tropical Indo-Pacific region. Indeed, video captured using a remotely operated vehicle at Rapa Nui (Easter Islands) reveals what appears to be another as-yet un-named species of banded *Prognathodes* inhabiting MCEs (Easton et al. 2017).

## Supplementary Material

XML Treatment for Prognathodes geminus

## Footnotes

Pyle and Kosaki (2016) incorrectly stated that the pelvic-fin spine of P.basabei was “shorter” than that of this species, although they correctly presented the corresponding proportional measurements.
